# A voluntary code of conduct for promoting adherence to the Mediterranean diet: rationale and propositions

**DOI:** 10.1017/jns.2026.10079

**Published:** 2026-07-09

**Authors:** Jacques Delarue, Sandro Dernini, Elliot M. Berry, Barbara Burlingame, María Marcela González-Gross, Roberto Capone, Yari Vecchio, Furio Brighenti, Antonia Trichopoulou, for the Joint Med Diet Task Force of International Center for Advanced Mediterranean Agronomic Studies (CIHEAM), Federation of European Nutrition Societies (FENS), International Union of Nutritional Sciences (IUNS)

**Affiliations:** 1 ER 7479 SPURBO, Faculty of Medicine, University of Brest, Brest, France; 2 International Center for Advanced Mediterranean Agronomic Studies (CIHEAM), Valenzano (Bari), Italy; 3 Braun School of Public Health, Hebrew University - Hadassah Medical School, Jerusalem, Israel; 4 Riddet Institute, Massey University, Palmerston North, New Zealand; 5 ImFINE Research Group. Department of Health and Human Performance, Faculty of Physical Activity and Sport Sciences-INEF, Universidad Politécnica de Madrid, Madrid, Spain; 6 Spanish Nutrition Society (SEÑ), Madrid, Spain; 7 Department of Veterinary Medical Sciences, University of Bologna-Alma Mater, Bologna, Italy; 8 Department of Food and Drug, University of Parma, Parma, Italy; 9 Hellenic Health Foundation, University of Athens, Athens, Greece

**Keywords:** Mediterranean diet, nutrition policies, public health and nutrition policies, sustainability

## Abstract

Throughout the Mediterranean region, adherence to the Mediterranean diet is decreasing especially among young people. The Mediterranean diet is the most studied dietary pattern with proven health benefits, especially regarding the prevention of non-communicable diseases at a time when their incidence is increasing worldwide. It has also been recognized as a sustainable diet model with multiple interdependent benefits on social, cultural, environmental, and economic dimensions. Faced with the challenge of promoting adherence to the Mediterranean diet, a Joint Med Diet Task Force of CIHEAM, FENS, and IUNS was formed to set the path for reversing the erosion of the Mediterranean diet heritage, by promoting its benefits, as a way of living, as defined by UNESCO. In this paper, the rationale and propositions of the Joint Task Force are described for the development of a voluntary code of conduct for promoting the adherence of the Mediterranean diet, and sustainable diets *per se*, addressed to all interested stakeholders and rights holders, linking food consumption and production, toward sustainable food systems transformation in the Mediterranean and beyond.

## Introduction: the problem of low adherence to the Mediterranean diet

The Mediterranean diet is a widely accepted healthy diet supported by scientific evidence from epidemiology and clinical trials, including randomized control trials, and systematic reviews and meta-analyses^(1–4)^. It is defined by the UNESCO in the inscription to the list of the Intangible Cultural Heritage of Humanity, as ‘*a set of skills, knowledge, practices and traditions ranging from the landscape to the table, including the crops, harvesting, fishing, conservation, processing, preparation and, particularly, consumption of food. The MedDiet is characterized by a nutritional model that has remained constant over time and space, consisting mainly of olive oil, cereals, fresh or dried fruit and vegetables, a moderate amount of fish, dairy and meat, and many condiments and spices, all accompanied by wine or beverage infusions, always respecting beliefs of each community. However, the MedDiet (from the Greek “diaita,” or way of life) encompasses more than just food. It promotes social interactions, since communal meals are the cornerstone of social customs and festive events’* ^(5)^.

The Mediterranean diet, recognized as a healthy dietary pattern^(6–8)^, is also acknowledged as a sustainable diet model, linking nutrition and sustainable food systems^(9–13)^.

Despite all its well-documented benefits, the Mediterranean diet is almost abandoned in most Mediterranean countries. Scientific evidence shows a tendency of Mediterranean populations to change their dietary patterns in favour of unhealthy dietary patterns^(14–17)^, and mainly among the young generation^(18–28)^. Some Southern and Eastern Mediterranean countries are still experiencing the ‘nutritional transition’, first characterized in the early 1990s^(29)^, in which problems of under-nutrition coexist with overweight, obesity and diet-related chronic diseases^(30)^.

The traditional ways of consuming and producing food in the Mediterranean area have changed considerably, due to economic, social, cultural, demographic, and technological trends, increasing globalization, urbanization, and shifting lifestyles with increased sedentary daily life^(11)^. The erosion of the Mediterranean diet is alarming, as it has undesirable impacts not only on health and nutrition, but also on social, cultural, economic, and environmental sustainability dimensions in Mediterranean countries^(12,13)^.

Current unhealthy and unsustainable food production and consumption patterns (fast and ultra-processed foods) have a strong negative impact on overall food systems sustainability^(31–33)^.

The globalization of eating habits has overtaken traditional diets and increased sedentary lifestyles, also throughout the Mediterranean region and beyond.

Studies on the Mediterranean diet were/are mainly focused on health/nutrition impacts (physiological needs for energy, and macro- and micronutrients) of its characteristic foods, and recently also on its environmental impacts, while the importance of its cultural, social and economic food dimensions have been rather neglected, despite being shared by all Mediterranean peoples^(34)^. In the last decade the notion of the Mediterranean diet has undergone a progressive evolution, from a traditional healthy dietary pattern to a sustainable diet model, a Mediterranean lifestyle^(35,36)^.

In people’s daily lives, however, things do not work this way as daily food choices are influenced by economic, political, social, cultural, and physical components, most of which are outside the control of the individual^(37,38)^.

## Why is the Mediterranean diet sustainable?

The Mediterranean diet, predominantly plant-based with low consumption of animal and industrial products, has been characterized as a sustainable diet case study with multiple interdependent sustainable benefits, with country-specific variations: (1) well-documented health and nutrition benefits; (2) low environmental impacts and richness in biodiversity; (3) positive local economic returns; (4) high socio-cultural food values, including dialogue among different cultural identities and food traditions, mutual respect and social inclusion^(39)^.

A sustainable diet that supports both individual well-being and environmental sustainability must also be economically accessible and culturally respectful to facilitate its widespread adoption and contribute to broader societal benefits^(40–43)^. Additionally, economic sustainability means that diets should be structured to avoid overburdening household finances, ensuring access to healthy food without compromising other essential needs^(44)^. The social aspect of sustainability necessitates that diets be inclusive and respectful of local food traditions, which is crucial for their long-term acceptance and adherence. In the case, promoting the adherence to the Mediterranean Diet model requires highly to consider the history of the Mediterranean^(45,46)^. In many communities, introducing dietary changes without respecting existing cultural practices can encounter resistance and fail to achieve public health goals^(47)^. Therefore, it is essential that food policies and dietary guidelines include the participation of local communities in their development. Another fundamental aspect of a sustainable diet is fair remuneration for producers^(48)^. From an ethical standpoint, sustainability cannot be achieved without ensuring that farmers, fishers, and small-holder producers receive fair compensation for their labour and products, as the core principle of sustainable consumption is rooted in ethically responsible food production, where producers are paid fairly, ideally through short supply chains and local farmer markets^(49)^.

It is therefore necessary to distribute the value equally along the entire supply chain and not only benefit the trade and industry sector as happens today.

Geographical accessibility is a critical factor, as both rural and urban areas must be served by efficient distribution networks to ensure the availability of healthy and sustainable food. Furthermore, the Mediterranean diet model promotes sustainable agricultural practices, which contribute to the conservation of biodiversity^(50)^ and sustainable management of natural resources^(51)^.

## Revitalizing the Mediterranean diet model

There are two competing schools of thought on how best to revitalize the Mediterranean diet. One position promotes the need for a more flexible approach to the Mediterranean diet to make it compatible with contemporary life, accommodating the influences of modern food processing and lifestyle changes. The other position posits that the Mediterranean Diet model of the 1960s is still largely possible, by practicing many of the lifestyle and dietary choices of the past and resisting many of the unhealthful and unsustainable choices of contemporary food systems. Regardless, the evidence-basis for both positions acknowledges that challenges posed by modern food environments, including the rise of ultra-processed foods, and that the solution requires a transdisciplinary narrative for the Mediterranean diet, as a model of sustainable production and consumption, overcoming the silos of specialization, and fragmented sectoral approaches. Policies and initiatives in the health sector need to converge with those in the agriculture and environment sectors, so that approaches are complementary and congruent^(42)^.

Cultural identity and family traditions play key roles in shaping food choices, often guiding individuals toward healthier, more sustainable practises, mixed with the ‘modern’ facets of social media, environmental consciousness, stigma and responsibility, coolness, perceived status, and satisfaction in the act of eating, especially in the company of family, friends and colleagues^(52–54)^.

Taste and emotions aroused by ‘traditional’ foods can be a compelling starting point for revitalizing the Mediterranean diet^(54)^. Nostalgia often leads to a preference for familiar, comforting foods, which can sometimes coincide with healthier food choices when linked to home-cooked, traditional meals. Some studies have linked traditional foods and culinary practises to healthier, more sustainable diets, particularly in the context of modern, fast-food-driven societies^(55)^.

Many lifestyle choices that resonate with aspects of individual responsibility, such as the protection of nature and the environment, fairness in work, animal welfare, etc., can find common ground on promoting the Mediterranean diet^(56)^.

The question remains: how best to revitalize the Mediterranean diet? Considering the challenges for reversing the erosion of the Mediterranean diet, a voluntary code of conduct is proposed as a case for action for sustainable diets, in the light of the current ongoing debate on ‘*sustainable diets*’^(11)^, ‘*sustainable healthy diets* ^(57)^, and *healthy diets* ^(58)^ in the context of a sustainable food systems *transformation* ^(59)^.

## Voluntary code of conduct—case for action

The rationale for co-developing the conceptual framework of the proposed Mediterranean diet voluntary code of conduct is based on the recognition that the challenges threatening the Mediterranean diet heritage are complex, interrelated, and context-specific. Addressing them requires a sustainable food systems approach grounded in broad, multi-stakeholder collaboration and consensus, rather than fragmented, sector-specific solutions^(59)^.

Mediterranean food systems and diets have become increasingly detached from local food production^(60)^. Given the intrinsic linkages among food production, consumption, and nutritional health, consumers occupy a pivotal role within food systems. Improving adherence to the Mediterranean diet requires the active involvement of consumers and all sectors, with regional cuisines, chefs and collective catering services playing an important educational role in fostering awareness and appreciation of local food biodiversity and the diversity of traditional foods and recipes^(61)^.

The incorporation of sustainability issues into food-based dietary guidelines has been steadily increasing over the past decades to make diets healthier for consumers as well as for the environment. However, this effort has not met universal approval. After the publication of the first dietary guidelines for sustainability^(62)^, criticisms have continued to ignite controversial debates, particularly in the agriculture sector^(63–65)^. Within this international debate on the transformation of sustainable food systems towards more sustainable dietary patterns, the value of the Mediterranean diet, a mainly plant-based dietary pattern, is acknowledged as a sustainable diet model^(66–69)^.

The Mediterranean diet model offers significant opportunities to preserve health, culture and biodiversity while improving environmental, socio-cultural, and economic sustainable benefits. However, in spite of all this evidence, its adherence is decreasing. Many barriers may explain this, such as taste, cost, convenience, culinary preparation skills^(70)^, time constraints, education, income, food environments, and the ubiquity of ultra-processed foods^(71)^, which have been shown to be harmful for health^(72)^. In 2004, the International Task Force on the Mediterranean Diet proposed ‘*the possibility of defining the Mediterranean diet with certain openness that would acknowledge healthy changes, within this model that may have been produced over last 40 years, or that may come about in the future’* ^(73)^. Such changes were expected to be beneficial for improving its adherence, without distorting its identity. For example, the Prevención con Dieta Mediterránea study provides evidence that a slight modification of the traditional healthy Mediterranean diet can still benefit people at high cardiovascular disease risk^(74)^.

Concerning affordability, a recent review concluded that the Mediterranean diet could be less costly than Western diets and could bring a beneficial cost-benefit for health systems^(75)^. Production intensification has also degraded agricultural landscapes, decimated ecosystem services, and contaminated foods and agri-food systems through excessive use of persistent and harmful agricultural chemicals, resulting in lower concentrations of nutritionally desirable compounds and/or higher concentrations of toxic compounds in many of the foods associated with the health benefits of the Mediterranean diet^(76,77)^. For most populations, including in Mediterranean countries, livestock systems are by far the main factors associated with deleterious environmental impact, and, for many people, reducing their meat consumption is not easy^(78)^. In the Spanish landscape called « Dehesa » the Iberian pork, sheep, and goat, contributes to sustainability and the environment, and probably in other Mediterranean countries it is similar.

The Mediterranean diet emphasizes the use of local and seasonal products, thus reducing the environmental impact of food transportation and storage. In addition, the cultivation of olive trees, vineyards, and other typical Mediterranean crops supports the local economy and preserves agri-cultural landscapes^(79)^. In the past four decades, several pyramids represented the Mediterranean diet dietary pattern characteristics, However, these studies vary considerably in definitions and in defining the amounts of foods in grams and/or nutrients constituting the model^(80)^. In 2020, through a consensus process, an updated version of the 2012 Mediterranean diet pyramid^(81,82)^, more focused on its environmental impact was published. In 2025, an adapted version of a Mediterranean Lifestyle Pyramid addressed to children and adolescents was developed, through a consensus process by another group of experts^(83)^.

## The code

The voluntary code of conduct, through broad consultation, is addressed to promote the *Mediterranean diet* model *by* underscoring its common principles^(84)^:

- Variety and balanced food combination: Different food, with more fruits and vegetables of diverse colours
- Seasonality: Fresh foods, minimally processed
- Traditional, local food products, biodiversity, agro-eco-friendliness: Territorial linkages—sustainable rural development
- Culinary activities: Preservation and transmission of food knowledge, skills, practises and heritage and pleasure of eating
- Conviviality: Pleasure of eating together—dialogues between people and cultures
- Frugality and moderation: Small portion sizes—major public health challenge of obesity—food has value, do not waste
- Active living: Physical activity—non-sedentary lifestyle

### Aims of the code

- To promote the adherence as well as the sustainability of the Mediterranean diet by enhancing scientific knowledge on its common principles.
- To safeguard the common principles of the Mediterranean diet heritage, as a model of sustainable diet, in different settings and through multiple channels, including hospitals, collective catering, schools, restaurants, and media.
- To serve as a resource of the Mediterranean diet common principles for scientists, public and private policy makers, and all other food system stakeholders, to improve the adherence and sustainability of the Mediterranean diet, at the country level.

The code will address the sustainability of the Mediterranean diet with the sustainable food systems approach of the SFS-MED Platform^(59)^, considering sustainable food systems as a whole, rather than their separate parts, and going beyond disciplinary approaches and silos, as a web of interconnected and interdependent components within a decision-making very fragmented food environment, with a wide range of voices from different interest groups and agendas^(59)^.

By taking into account this interdependent food environment, following cross-cutting domains, were initially identified:

1. Nutrition and health
2. Sustainable diets
3. Sustainable food systems
4. Environment
5. Agriculture
6. Fisheries and aquacultures
7. Food industries
8. Retail distribution
9. Food services, catering, restaurants
10. Food environment, consumer science
11. Food loss and waste
12. Economics
13. Research, innovation, capacity building
14. Education and training
15. Media
16. Digital technologies and artificial intelligence
17. Implementation science

These cross-cutting domains will be approached through a whole adaptive sustainable food systems perspective^(85,86)^ to further understand their interconnectivity and how multifaceted factors across systemic levels can play a major role in the revitalization of the Mediterranean diet as a sustainable diet model, contributing to improve in the region food security and environmental, economic and social sustainability^(66)^.

The code will begin with a preamble setting out the general justification. The preamble of the *International Code of Marketing of Breast-milk Substitutes* ^(87)^ has been used as a model for a sustainable diets’ code of conduct preamble, which in turn is applicable to the Mediterranean diet.

For the purpose of this exercise, the 2010 FAO definition of sustainable diets^(11)^ has been modified and used as the *ad hoc* definition of the Mediterranean diet, as follows:

‘…*is a diet with low environmental impacts that contributes to food and nutrition security and to healthy life for present and future generations in the Mediterranean region. It is protective and respectful of biodiversity and ecosystems, culturally acceptable, accessible, economically fair and affordable; nutritionally adequate, safe and healthy; while optimizing natural and human resources*’.

This definition is not to be construed as an official, proposed, or suggested definition for the Mediterranean diet, simply is being used as a surrogate or proposal for the specific purpose of this drafting exercise of the voluntary code of conduct.

**Implementation process of the development of the voluntary code—how and by whom**

- Enhancing a broad consensus process
- Considering the centrality of the food environment
- Considering the bio-psycho social and sociotype
- Developing a unique Mediterranean diet adherence score
- Coping with barriers to the Code’s development
- Development of the zero draft of the code and its action plan

### Enhancing a broad consensus process

In 2010, a consensus definition for sustainable diets was reached at the International Scientific Symposium ‘*Sustainable diets and biodiversity*: *United against hunger*’, by FAO and Bioversity International at the FAO headquarters, in Rome, in which an entire session was devoted to the Mediterranean diet as an example of a sustainable diet, and closed with a proposal for drafting a code of conduct for sustainable diets^(11)^. In 2011, at the FAO/CIHEAM International Workshop *‘Guidelines for the Sustainability of the Mediterranean Diet’,* at the CIHEAM-Bari, was started as a joint case study to assess the sustainability of the Mediterranean diet in the Mediterranean region, considering its multi-dimensions and impacts on health and nutrition, environment including biodiversity, economy, and socio–cultural factors^(88,89)^. In 2016, at the first World Mediterranean Diet Conference ‘*The Revitalizing the Mediterranean Diet: from a healthy dietary pattern to a healthy Mediterranean sustainable lifestyle’* ^(90)^, **‘** *The Call for Action on the Revitalization of the Mediterranean Diet’* was issued and endorsed by 35 international and national scientific institutions^(91)^.

In 2017, this long-standing consensus process continued at the FAO/CIHEAM international workshop ‘*Development of voluntary guidelines for the sustainability of the Mediterranean diet in the Mediterranean region*’, held at the CIHEAM Bari^(84)^. In 2019, at the Second World Mediterranean Diet Conference ‘*Strategies toward more sustainable food systems in the Mediterranean region: the Mediterranean Diet as a lever for bridging consumption and production in a sustainable and healthy way*’ held in Palermo by CIHEAM-Bari and the Forum on Mediterranean Food Cultures^(92)^, the issue of a voluntary code of conduct for the sustainability and adherence of the Mediterranean diet was raised again.

In 2022, at the Third World Mediterranean Diet Conference ‘A c*hange of route: towards more sustainable and resilient food systems in the Mediterranean countries - The Mediterranean diet as a strategic resource for accelerating the Agenda 2030 in the Region*’^(93)^, by CIHEAM-Bari, as an outcome of the session ‘*Assessing and promoting adherence to the Mediterranean diet’*, a Joint Med Diet Task Force was initiated by FENS, IUNS Sustainable Diets Task Force, and CIHEAM-Bari to set the path for reversing the erosion of the Mediterranean diet, as a way of living in the Mediterranean.

The role of the Task Force was setting the path for creating, evaluating, and implementing a common framework for a new Mediterranean Dietary Pattern for the 21st Century, by

- (re-)defining the Mediterranean Diet appropriate for the 21st Century;
- reaching consensus, on the elements required to measure adherence to MedDiet, and, if possible, developing a single index;
- drafting a voluntary code of conduct with recommendations for different sectors (primary producers, food industry, health, environment, food service and consumers, etc.) to improve adherence to sustainable Mediterranean Diets, using a rights-based approach, grounded in tradition, respectful of biodiversity and ecosystems, and consistent with Sustainable Development Goals.

In November 2023, at the 14th European Nutrition Conference FENS 2023, in Belgrade, the proposal of the voluntary code of conduct was further discussed within the double symposium ‘*Measuring and Promoting Adherence to the Mediterranean Diet, as a Model for Sustainable Diet’,* organized by the Joint Med Diet Task Force and supported by CIHEAM-Bari.

In April 2024, a first Med Diet Joint Task Force’s meeting in person was organized by CIHEAM Bari, in Rome, with the scope to work more efficiently on the Task Force’s objectives, updating its ToRs and defining its road map 2024-2025 towards the development and implementation of a zero draft of a voluntary code for sustainable diets, adapted to the Mediterranean diet.

In August 2025, at the IUNS/ICN 2025 conference, in Paris, an overview update on the proposal development of the voluntary code of conduct was debated, within a dedicated symposium on ‘*A voluntary code of conduct for measuring and promoting adherence and sustainability of the Mediterranean diet:- rationale, proposition and challenges*’, organized by the Joint Med Diet Task Force, The symposium was focused to set a path for reaching consensus on a voluntary code of conduct and a unified score, as safeguarding measures, to reverse the erosion of the Mediterranean diet by promoting and measuring its adherence and sustainability. The symposium ended by discussing next steps on how to lead the development and implementation process of the code as a broader collaborative effort, involving expertise from other fields not represented yet in the Task Force.

### Considering the centrality of the food environment

Food behaviours and choices of consumers are linked to the food environment^(94–96)^, that is ‘*the physical, economic, political and socio-cultural context, in which consumers interact with the food system to make their decisions on acquisition, preparation and consumption of foods*’^(13)^.

The creation of food environments more ‘favorable’ to consumers to practise more sustainable food choices contributes to generating a *‘new food demand’* which, in turn generates a more sustainable *‘new food supply’*, which could have significant impacts on the transition towards more sustainable food systems and diets^(13)^. Sociocultural factors have a great influence on eating patterns, culture differentiates the way of eating and the relationship with food which can be ritual, habitual, symbolic, religious, or ethnic. But these variables that influence dietary change depend in turn on global socioeconomic change, that is, on population, the degree of economic development and international trade. In the scenario described the role that the Mediterranean consumer assumes, and will assume in the immediate future, is of priority importance for moving towards more sustainable food systems since sustainable consumption and production in the agri-food sector is a holistic concept oriented by consumer^(94)^. It refers to the integrated implementation of sustainable models of food consumption and production, respecting the carrying capacity of natural ecosystems. It therefore requires consideration of all aspects and stages of a product’s life, from production to consumption, and includes issues such as sustainable lifestyles, sustainable diets, food losses and food waste management and recycling, voluntary sustainability standards and behavioural environmentally friendly methods that minimize negative impacts on the environment and do not jeopardize the needs of present and future generations. For this reason, giving greater emphasis to the consumer has an enormous potential to impact the sustainability of food systems^(94)^. The role of the consumer therefore becomes crucial in the co-creation of the sustainability model. Through their choices, such as purchasing local products, they close the circle for the co-creation of the food environment. Creating ‘sustainable’ food environments ultimately means ensuring that the foods, drinks and meals that contribute to healthy and sustainable diets are the most available, accessible, convenient, and enjoyable^(97)^. Actions that identify ‘food entry points’ can transform the structural factors that guide food choice, within the existing food environment in which citizens make their food choices^(98–101)^. On this aspect, a little field example is the effect of the arrangement and information of foods and drinks sold in vending machines at the University of Parma, which resulted in an increase in the purchase of healthier foods, without limiting the freedom of consumers^(102)^.

### Considering the bio-psycho social and sociotype

The bio-psycho social and sociotype approach should also be considered^(103)^. The Sociotype framework was developed as a summary ecological construct to organize the multiple, dynamic, reciprocal inputs from the environment that interact with the genotype (DNA), to determine the expression of observable individual phenotypic behaviours such as coping with life stresses. It has three domains—Individual; Relationships and Social Environment; and Institutional Context^(104)^. The sociotype approach recognizes that the consumer is not just a passive recipient of food but, rather, is actively involved in food choices at each domain, based on individual preferences, and family and social-economic circumstances. Traditional culinary knowledge will influence menu selections, while cultural traditions are reflected in the meals celebrating birthdays, weddings, religious and other festive occasions. The Sociotype framework can also present operational insights related to issues around food security and sustainable food systems, in particular regarding adherence to, and resilience of the Mediterranean diet and lifestyle^(105)^. Additional topics covered include the impact of crises on general food supply and implications for policy, strategies involving women as well as men (households) to overcome food insecurity, consumers’ attitudes and knowledge with respect to sustainable fisheries and fish consumption, and a potential Mediterranean diet curriculum for schools through food and nutrition education, with a focus on responsibility, frugality, creativity, and enjoyment. These concepts may be translated into *policy decisions* to promote adherence to the Mediterranean Diet model as part of complex adaptive sustainable food systems^(106)^.

### Developing a unique Mediterranean diet adherence score

Assessment of adherence to the Mediterranean diet is at the core of the adoption and application of the proposed Code of Conduct. To date, there exists a multitude of scores all aiming to evaluate dietary intake and how closely an individual’s diet matches the main elements of the Mediterranean diet. Currently, the multiplication of indexes of adherence^(80)^ some of them being adapted to consider local habits and traditional components of the Mediterranean diet, are an obstacle both to transfer the knowledge about health effects and to achieving sustainability for better adherence^(1,107,108)^. It is noteworthy that almost all indexes proposed include *only food aspects* of Mediterranean diet and not its other socio-cultural dimensions, acknowledged by UNESCO in 2010^(5)^.

Promoting adherence to the Mediterranean diet implies discontinuing the use of, and, *a fortiori,* to continue to develop so many Mediterranean diet Scores (MDS), which result in confusion, a lack of clarity and, in some cases, a wrong description of what is the Mediterranean diet. The available scores present multiple methodological and conceptual differences, ranging from what food components and lifestyle factors are included in the score, to scoring techniques and statistical evaluations. These differences often limit the comparability of findings across studies, settings, populations, and cultures. In this context, the Joint Task Force is proposing, in a second paper, the development of a unified global score (Unified Mediterranean Diet Score) that measures adherence to the Mediterranean diet^(109)^ considering: (a) inconsistency in food items across the scores; (b) lack of a comprehensive lifestyle approach with mainly focus on food-based components in the scores; (c) limited cultural specificity in Mediterranean Diet Scores (MDS), mainly originated from a regional focus by economically developed countries, (d) common food components across MDS; (e) different algorithms used in calculating MDS^(109)^.

It is important to note that such a unified Mediterranean diet score will not replace the existing scores, rather it is meant to complement them, adding a dimension to support the development of a global level code for sustainable diets.

### Coping with barriers to the Code development

In the Mediterranean region, there are different economic and social disparities made evident by the analysis of macroeconomic indicators which highlight a great heterogeneity between countries and an increasingly growing gap between developed economies and those that are less so. This leads to inevitable repercussions on the economic, social, environmental, and nutritional dimensions of the populations living in the region. In particular, the change in food consumption patterns, as well as economic factors, and therefore the ability to consume, are determined by the ability to produce and the ability to exchange. Sociocultural factors are also of great importance as food choices, given the same economic conditions, can be different depending on their belonging to different cultural models. But these variables that influence dietary change depend in turn on global socioeconomic change, that is, on population, the degree of economic development and international trade. A further element to consider is represented of multi-factor impacts of the consumer and of the Mediterranean consumer of the future. Inequalities barriers lead us to think that there are also differences in terms of food consumption, marked by the different ability to consume. Nutrition is a complex social fact, based above all on satisfaction, that is, on the satisfaction of hunger, but also on the pleasure of the palate, on the consumption of symbols and social signs. It, therefore, varies considerably from one social group to another. Within this society, lifestyles will be increasingly characterized by great speed in carrying out daily activities, different social strata in terms of gender, age, culture, and traditions^(94)^.

Within the United Nations system and its member states, codes of conduct are notoriously difficult to usher through, particularly when they require involvement of multiple sectors and disciplines for coping with interdependent barriers facing a sustainable food systems transformation^(59)^. However, for a voluntary code, as in the case of the Mediterranean diet, the process may be simpler by considering the well-established acknowledgement of its benefits, as well as, the threat of the growing erosion of its adherence among Mediterranean populations.

Regardless of the mechanism, the urgency for action associated with promoting adherence to the agreed elements of the Mediterranean diet cannot be overstated. The proliferation of ultra-processed foods, and the accompanying effects on human and planetary health, have sharpened the focus on the imperative for ‘code’ for a restoration of the Mediterranean diet, as a sustainable diet model for Mediterranean populations, in the context of the sustainable food systems transformation in the region.

Globally, and over many decades, hundreds of agreed texts in the form of guidelines, goals, targets, treaties, codes of conduct, declarations, action plans, and recommendations covering a variety of topics, have been produced. There is a long history of failures and unintended consequences in all sectors’ approaches, including interventions of one sector undermining those of the other. International initiatives and guidelines in nutrition, as well as those addressing environmental sustainability have largely been sector specific. For many decades, the nutrition and agriculture sectors were focused on dietary energy supply and food security.

### Development and finalization of the zero draft of the code and its action plan

The zero draft of the code of conduct, initiated in May 2024, after the Task Force meeting in Rome, will be further developed to be debated in 2026, at a high-level international conference, with the scope to be endorsed, through a broader consensus, by all stakeholder participants, towards its validation in potential interested Mediterranean countries.

In this *Voluntary code of conduct for promoting the adherence and the sustainability of the Mediterranean Diet,* the basic unit of nutrition, would not be individual nutrients, nor would it be food *per se*. It would have to be ‘diet’ and it would have to be addressed through an ecosystem approach to ensure nutrition and sustainability^(110)^.

This voluntary code of conduct on the Mediterranean diet, as an expression of the Mediterranean diet interconnections between different physical and cultural systems, such as the Mediterranean, can provide useful recommendations for more in-depth observations and analyses also for other territorial diets, in other *agroecological zone constraints* ^(111)^.

By incorporating the principles of the Mediterranean diet and its multiple health and sustainability benefits, a broader consensus process for the development of the voluntary code of conduct will facilitate, through different food environments, various channels, its operationalization, as a sustainable consumption and production (SCP) driver in the Mediterranean region.

## Conclusion

The loss of adherence to the Mediterranean diet in the region where it was born, requires urgent action for its revitalization by promoting its adherence and sustainability. In order to reach this objective, the setting of a ‘Mediterranean diet voluntary code of conduct’ was identified, since 2010, as an operational safeguard measure, across different interdependent sectors, for all stakeholders interested to the revitalization of the Mediterranean diet and in redefining its model appropriate for the 21st century.

The notion of the Mediterranean diet has progressively evolved over the past half a century, from a healthy (coronary) dietary pattern to a model of sustainable diet [35]. This evolution has transformed the whole concept of the Mediterranean diet from strictly medical and nutritional positions to visions more closely linked with society, culture, lifestyles, economy, sustainability, and environment.

These transformations have involved more and more professionals from different disciplines (medicine and nutrition, but also environmental studies, biology, food studies, social anthropology, sociology, economy, etc.) who have contributed their diverse points of view on a subject that, initially, was created with only health in mind.

Interdisciplinarity became an unavoidable part of the multidimensional analysis of the Mediterranean diet that needs more cross-disciplinary views in the development of the code. A multistakeholder and interdisciplinary implementation of the voluntary code of conduct will be of primary importance to build the necessary trust and commitments, based on shared understanding and inclusion for the success of the code endeavour.

The cultural, social, and economic reality of its protagonists need to be taken into account to improve adherence to the Mediterranean diet model. The inclusion of interdisciplinary perspectives and their interpretation will enrich the code, allowing future innovative and creative collaborations between different disciplines, within the highly changing and evolving Mediterranean diet sociocultural environment^(112)^.

Adopting a complex systems approach^(85,86)^ for the revitalization of the Mediterranean diet within the global food system is of paramount relevance as this would help further understand the interconnectivity of food systems and diets and how multifaceted factors across systemic levels will play a major role in the development and implementation of the voluntary code of conduct and its action plan for improving its adherence, and improving food security and sustainability at the country level. In this context, the SFS-MED Platform can play also a major role in amplifying the consensus process and its implementation.

The next step is bringing together more experts from other fields, (such as anthropology, consumer science, education, agriculture and fisheries, food industries, distribution, food services, education, media, digital technologies, etc.), towards a joint development of the code and its action plan, to be brought then to a broader spectrum of stakeholder and policy makers who have the power to operationalize it.

## AI

The authors have used no AI to write this paper.
